# The pathogenesis of hepatic steatosis in MASLD: a lipid droplet perspective

**DOI:** 10.1172/JCI198334

**Published:** 2025-09-16

**Authors:** Natalie Krahmer, Tobias C. Walther, Robert V. Farese

**Affiliations:** 1 Institute of Diabetes and Obesity, Helmholtz Munich, Neuherberg, Germany.; 2 German Center for Diabetes Research, Neuherberg, Germany.; 3 Cell Biology Program, Sloan Kettering Institute, Memorial Sloan Kettering Cancer Center, New York, New York, USA.; 4 Howard Hughes Medical Institute, New York, New York, USA.

## Introduction

Metabolic dysfunction–associated steatotic liver disease (MASLD) is closely linked to the global rise in obesity and affects approximately one in three adults worldwide. With morbid obesity, approximately 90% of individuals exhibit evidence of hepatic steatosis. In most individuals, MASLD is asymptomatic and benign, characterized by the accumulation of lipids in hepatocyte lipid droplets (LDs), dynamic organelles specialized for neutral lipid storage. However, a subset of patients progress to metabolic dysfunction–associated steatohepatitis (MASH), which can lead to fibrosis, cirrhosis, liver failure, and hepatocellular carcinoma. Current treatments for MASLD show promise but are only effective in a subset of patients, and no approved therapies directly target or reverse liver fibrosis.

The magnitude and pathological consequences of the MASLD epidemic and the relative lack of therapies underscore the need to better understand its molecular pathogenesis. The key question is how hepatic LD accumulation drives disease progression and what factors modulate its course. This Viewpoint synthesizes our current understanding and presents insights, grounded in LD biology, to propose a mechanistic model of MASLD pathogenesis.

## The origin of the problem: hepatocyte neutral lipid overload

The pathological accumulation of lipids within LDs of hepatocytes is a defining feature of MASLD. LDs are specialized organelles for neutral lipid storage that form through phase separation of lipids. They have a hydrophobic core made up of primarily triglycerides (TGs) and cholesterol esters (CEs), enclosed by a phospholipid monolayer harboring distinct proteins that help orchestrate lipid metabolism. Under pathological conditions, hepatocyte LDs can expand greatly in size, giving rise to macrovesicular steatosis, a key pathological and diagnostic feature of MASLD. This intracellular lipid overload can lead to cellular lipotoxicity, organelle dysfunction, and progressive liver injury.

The fatty acids that give rise to lipid accumulation in hepatocytes originate from two principal sources: exogenous fatty acids, derived from dietary intake and adipose tissue lipolysis, and endogenous fatty acids produced intracellularly via hepatic de novo lipogenesis (DNL). Under normal conditions, DNL contributes approximately 10% of hepatic FA input, but in obese and insulin-resistant states, this fraction may increase to approximately 40% (1).

Within hepatocytes, fatty acids are esterified into TGs by acyl-CoA:diacylglycerol acyltransferases (DGATs) or into CEs by sterol *O*-acyltransferases (SOATs). DGAT1 and DGAT2 are linked to different fatty acid sources: DGAT1 primarily esterifies exogenous fatty acids, and DGAT2 preferentially utilizes fatty acids generated by lipogenesis (2). Exogenous fatty acids utilized by DGAT1 must be “activated” to form acyl CoAs by acyl-CoA synthetases, whereas DGAT2 can utilize acyl-CoAs produced directly by DNL. Notably, DGAT2 activity appears to influence lipogenesis itself. DGAT2 inhibition or deletion reduces lipogenesis flux, whereas DGAT2 overexpression increases lipogenesis, suggesting feedback between TG and fatty acid biosynthesis (3–5).

CEs also accumulate in MASLD, though to a lesser extent than TGs. In human hepatocytes, the relative contributions of CE synthesis enzymes SOAT1 and SOAT2 to CEs are unclear but potentially important (6). In addition to esterified cholesterol, unesterified cholesterol can accumulate in hepatocytes, where it may crystallize and promote toxicity. For instance, disrupted cholesterol sensing through mutations in liver X receptor-α (LXRα) leads to accumulation of free cholesterol and may be a contributor to hepatocyte injury and liver dysfunction (7).

## Exceeding the lipid storage capacity of hepatocytes as a possible trigger for cell dysfunction

Neutral lipid accumulation leads to the formation of LDs in the ER of hepatocytes, mediated by LD assembly complexes (LDACs), which consist of the proteins seipin and LDAF1 (8). These LDs are usually less than a micron in diameter in cultured cells, but larger droplets of a few microns in diameter are occasionally seen. In MASLD, LDs are dramatically enlarged to many microns in diameter, resulting in the characteristic histological pattern of macrosteatosis. Moreover, the size distribution of LDs in MASLD hepatocytes is broader than that in cultured cells, indicating a dysregulation in LD formation and growth.

A central pathogenic feature in MASLD may be the limited capacity of hepatocytes to efficiently form and expand LDs. Hepatocytes, unlike most cells, not only store neutral lipids but also secrete these lipids as components of apolipoprotein B–containing lipoproteins. Thus, hepatocytes may have evolved to reduce intracellular storage machinery (i.e., LDACs) in the ER to enhance the capacity for neutral lipid secretion toward the ER lumen. Supporting this, hepatocytes express relatively lower levels of seipin and LDAF1 than other cells, such as adipocytes, potentially constraining LD biogenesis capacity.

If hepatocytes indeed have a lower “ceiling” for TG storage, they may be more easily overwhelmed. When the rate of TG synthesis in the ER exceeds the cell’s capacity to generate and expand LDs, neutral lipids may aberrantly phase separate from the ER, akin to the disorganized LD phenotype in seipin-deficient cells, characterized by LD heterogeneity and a tendency toward fusion (9). Thus, a physiological trade-off between neutral lipid storage in LDs and secretion in lipoproteins could render hepatocytes particularly vulnerable to TG overload and storage in metabolic disease.

## A potential role for phospholipids and packing defects in MASLD

What drives the formation of abnormally large LDs in MASLD? One potential mechanism involves a deficiency in phospholipids, particularly phosphatidylcholine (PC), the major phospholipid coating LD surfaces. PC synthesis occurs in the ER, and hepatocytes must produce PC for assembly and secretion of lipoproteins. If the needs for PC synthesis are overwhelmed (e.g., in the setting of high TG synthesis and demand for LD expansion), limited PC availability may impair LD stability (10, 11). Without adequate PC, the surface tension of LDs is increased, leading to fusion of smaller LDs with larger LDs, thereby reducing the total surface area, a hallmark feature of macrosteatosis. LD instability may be further exacerbated by disruptions in phospholipid metabolism that shift the balance toward conically shaped phosphatidylethanolamine (12), which is less effective than cylindrical PC in reducing surface tension. Limited PC availability when LDs form can also reduce lipoprotein secretion. Thus, the retention of excessive fatty acids in hepatocytes can result in a vicious cycle of TG storage and secretion defects.

Experimental evidence supports that PC deficiency contributes to MASLD by destabilizing LDs. In vitro and in cells, surface packing defects that expose the neutral lipid core in PC-deficient or cone-shaped phospholipid-enriched LDs promote LD coalescence (11). While in nutrient-rich conditions, cells typically triple PC levels to accommodate increased TG storage (10), liver biopsies from patients with MASLD show elevated TG without a corresponding rise in PC, altering the TG/PC ratio and compromising LD stability (13). Similarly, PC-deficient mouse models (e.g., CTα or PEMT knockout mice) develop steatosis (14, 15) and rodent MASLD/MASH models often rely on choline-deficient diets, which limit PC synthesis and contribute to fat accumulation.

Normally, a PC-rich monolayer and LD-associated proteins stabilize the LD surface and prevent inappropriate protein binding. Without sufficient PC, amphipathic α-helical proteins mislocalize to LDs, impairing cellular functions. For example, conditions of elevated TG/PC levels lead to mislocalization of Golgi apparatus proteins to LDs and disrupted vesicular trafficking and secretion, impairing lipoprotein export (15, 16). Additionally, PC deficiency activates stress pathways and contributes to mitochondrial dysfunction, altered membrane signaling, loss of hepatocyte identity, and metabolic imbalance, all hallmarks of advanced MASLD.

## LD protein variants and MASLD risk

Although obesity is the primary driver of MASLD, genetic factors — in particular those involving LD proteins — strongly influence individual susceptibility. The I148M substitution in the lipase PNPLA3 is the strongest known genetic risk factor for MASLD (17). Although PNPLA3 is a paralog of adipose TG lipase (ATGL), it exhibits minimal TG hydrolase activity, and the physiological function of the enzyme is poorly understood. The I148M variant is more stable, accumulates on LDs, and interferes with ATGL-mediated lipolysis by sequestering CGI-58, an essential ATGL coactivator (18). Overall, PNPLA3 variants may predispose to the formation of enlarged LDs, increasing the TG-to-PC ratio and possibly exacerbating lipid export defects.

Hydroxysteroid 17-β-dehydrogenase 13 (HSD17B13) is a LD-localized member of the short-chain dehydrogenase/reductase family. Multiple loss-of-function variants of it lead to inactive forms of the enzyme, associated with a reduced risk of progression to MASH and hepatocellular carcinoma (19). Although its endogenous substrate is unknown, HSD17B13 is thought to act on lipid-derived inflammatory intermediates, implicating it in liver inflammation and injury.

Cell death–inducing DFFA-like effector B (CIDEB) is an ER- and LD-associated protein that is implicated in TG mobilization from LDs to secreted lipoproteins. Paradoxically, rare germline loss-of-function and missense variants in human *CIDEB* are associated with lower risk of liver disease, including cirrhosis, suggesting a protective effect (20) by preventing excessive LD expansion.

## The potential consequences of MASLD

The accumulation of excess fatty acids in hepatocytes can result in downstream consequences affecting the metabolism and LD biology of other hepatic cell types. Stressed hepatocytes may release proinflammatory cytokines and exhibit damage-associated molecular patterns that activate immune cells, such as macrophages and stellate cells, triggering extracellular matrix secretion, inflammation, fibrosis, and ultimately cirrhosis. While stellate cell biology has been reviewed in detail elsewhere, the role of LDs in these cells during MASLD is poorly understood. Activation of stellate cells is associated with loss of retinyl ester storage in LDs and the release of transcriptionally and bioactive retinoids that activate profibrotic signaling (21, 22). Concurrently, lipid-associated macrophages, characterized by TREM2 expression and LD, accumulate around steatotic hepatocytes. These macrophages support collagen degradation and lipid clearance. Trem2-deficient mice exhibit worsened liver inflammation and injury, while TREM2 overexpression enhances lipid handling and reduces liver damage in mice (23).

## Implications for MASLD treatment

If lipid accumulation in hepatocytes is a primary inciting factor in MASLD pathogenesis — much like lipid accumulation in the arterial wall initiates atherosclerosis — then reducing hepatic lipid burden becomes a central therapeutic goal. This strategy targets the root cause of downstream cellular dysfunction, inflammation, and fibrosis.

Currently, glucagon-like peptide-1 (GLP-1) receptor agonists are the most promising approved pharmacologic class for alleviating MASLD. These agents reduce body weight, improve insulin sensitivity, and secondarily reduce hepatic steatosis (24). Since the GLP-1 receptor is not expressed in the liver, these effects may predominantly be mediated by systemic weight loss. Resmetirom, the first FDA-approved drug for MASH and a liver-specific thyroid hormone receptor agonist (25), also targets hepatic lipid metabolism.

Several emerging therapeutic strategies directly target hepatic lipid metabolism. DGAT2 inhibitors, for instance, reduce TG synthesis and, based on multiple studies, suppress lipogenesis, likely via feedback inhibition of sterol regulatory element-binding protein 1 activity. DGAT2 inhibitors have shown promising results in clinical trials (26, 27). Acetyl-CoA carboxylase (ACC) inhibitors reduce lipogenesis but may also induce compensatory hypertriglyceridemia (28). To leverage the positive effects of both inhibitors, a combination of DGAT2 and ACC inhibitors is being tested (28); this approach may also help counteract lipotoxicity by reducing ER stress and lipid burden.

Additional treatment strategies emerge from the lipid-centric model of MASLD, although these are largely conceptual at this point. LD-associated proteins, such as PNPLA3 and HSD17B13, are major genetic determinants of MASLD. Their precise molecular functions remain incompletely defined, but they represent potential targets for future therapeutic strategies: PNPLA3 by preventing its accumulation on LDs, and HSD17B13 by inhibiting its enzymatic function. Another strategy may involve increasing PC biosynthesis to ensure proper LD surface coating and prevent coalescence; potential approaches could include targeting the CDP-choline pathway or supplementing dietary choline, though the efficacy of such interventions in humans is uncertain. Enhancing fatty acid oxidation could provide an alternative disposal route for surplus lipids, but the optimal means to selectively boost hepatic oxidation without inducing mitochondrial stress or ketogenesis is yet to be fully defined. Inhibiting ACC1, ACC2, or ATP citrate lyase (ACLY), upstream drivers of DNL, has shown promise in preclinical models. Notably, the hepatic ACLY inhibitor bempedoic acid may lower liver TG levels through mechanisms independent of direct ACLY inhibition (29). Ferroptosis has been implicated in MASLD, and LDs may sequester polyunsaturated fatty acids in TG and prevent lipid peroxidation and hepatocyte injury.

Preventing hepatic stellate cell activation, potentially by preserving retinyl ester–rich LDs or blocking their lysosomal degradation, also presents a rational antifibrotic strategy, though the feasibility and specificity of this approach is unknown. These findings underscore the need for further research to elucidate the precise effects of these agents on hepatic lipid flux and systemic lipid metabolism.

Overall, a mechanistically grounded understanding of LD biology in MASLD offers a framework for therapeutic innovation, but many key details, especially regarding efficacy and safety of specific approaches, must still be determined.

## Conclusion — a LD-based model for MASLD progression

**Figure 1:**
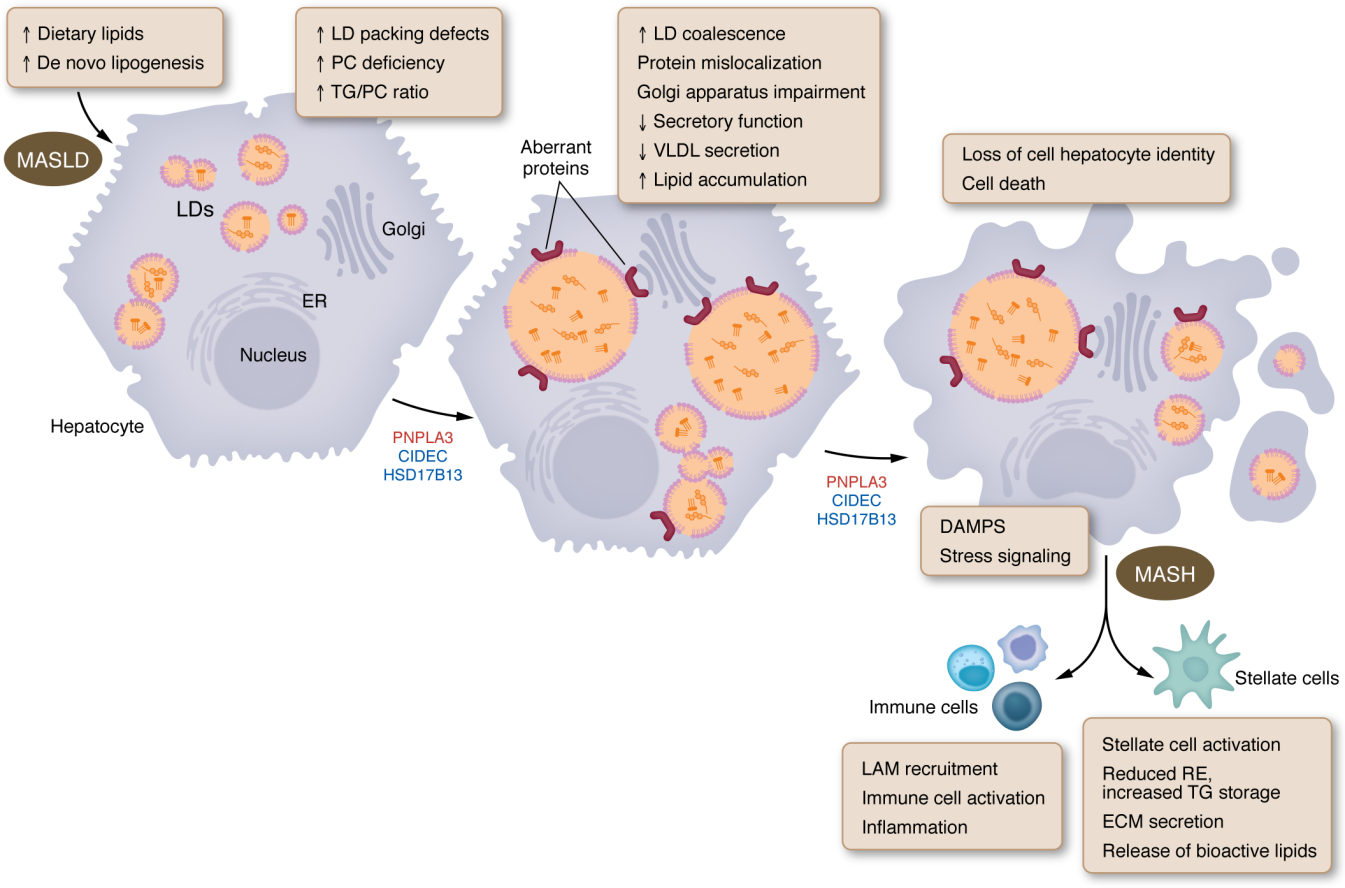
Model for involvement of lipid droplets in MASLD. Dietary lipids and increased de novo lipogenesis drive lipid droplet accumulation. A deficiency in PC and accumulation of phospholipids with poor surfactant properties result in unstable LDs with packing defects. These defects promote spontaneous LD coalescence and formation of giant LDs. Such aberrant LDs recruit non-LD proteins, including Golgi apparatus components, disrupting essential cellular processes, such as lipoprotein secretion. This exacerbates steatosis, activates stress pathways, impairs hepatocyte identity and function, and contributes to cell death. Stressed and dying hepatocytes release damage-associated molecular patterns and other signals that recruit lipid-associated macrophages and activate immune cells and hepatic stellate cells, leading to inflammation, fibrosis, and potentially cirrhosis or hepatocellular carcinoma. Genetic variants in LD-associated proteins (e.g., PNPLA3, HSD17B13, and CIDEB) can either promote (red) or protect (blue) against hepatocyte lipid accumulation, thereby influencing the risk and progression of different stages of liver disease. LD, lipid droplet; TG, triglycerides; PC, phosphatidylcholine; RE, retinyl ester; ECM, extracellular matrix; DAMPS, damage-associated molecular patterns; LAM, lipid associated macrophages.

This Viewpoint integrates current research to propose a molecular model for MASLD pathogenesis through the lens of LD biology (Figure 1). In hepatocytes, the convergence of excessive TG accumulation, the buildup of additional lipotoxic lipid species, such as cholesterol or fatty acids, impaired LD biogenesis, and the relative PC deficiency, may underlie the transition from simple steatosis to hepatocellular dysfunction. The disruptions in cellular LD architecture and function can lead to organelle stress, protein mislocalization, and activation of inflammatory signaling pathways. In parallel, the loss of retinyl ester-LDs (RE-LDs) by stellate cells is a hallmark of their activation. This shift reflects a metabolic reprogramming that transforms quiescent stellate cells into profibrogenic myofibroblasts and contributes directly to fibrosis and disease progression. Further studies are needed to dissect these lipid storage pathways in both cell types in greater detail to clarify causality, define regulatory nodes, and identify therapeutic strategies that may mitigate MASLD and its progression to MASH and fibrosis.

## Version 1 09/16/2025

Electronic publication
